# Association between Height and Actinic Keratosis: A Nationwide Population-based Study in South Korea

**DOI:** 10.1038/s41598-018-29155-6

**Published:** 2018-07-18

**Authors:** Young Bok Lee, Ji Hyun Lee, Min Ji Kang, Jin-Wou Kim, Dong Soo Yu, Kyung Do Han, Yong Gyu Park

**Affiliations:** 1 Department of Dermatology, College of Medicine, The Catholic University of Korea, Seoul, Korea; 2 Department of Biostatistics, College of Medicine, The Catholic University of Korea, Seoul, Korea

## Abstract

The association between actinic keratosis (AK) and anthropometric measures has not been investigated. This study aims to evaluate the associations between anthropometric measures and the incidence of AK in South Korea. We analyzed clinical data from individuals aged over 20 years who received a health examination arranged by the national insurance program between 2005 and 2008. Newly diagnosed AK was identified using claims data from baseline to the date of diagnosis or until December 31, 2015. The incidence of AK was highest among the elderly (aged over 60 years) and showed a male bias. The risk of AK increased with greater height. The quintile with the greatest height had an increased risk of AK compared with the quintile with the lowest height (hazard ratio = 1.28, 95% confidence interval: 1.24–1.33) after adjustment for age, sex, income, smoking status, alcohol consumption, hypertension, dyslipidemia, myocardial infarction, congestive heart failure, and chronic obstructive pulmonary disease. This study showed a positive association between the incidence of AK and human height. However, it is unclear whether these findings can be generalized to Koreans who have not received an examination or to populations in other countries.

## Introduction

Actinic keratosis (AK) is a common skin disorder composed of proliferative and transformed keratinocytes. AK is characterized by rough patches or papules with erythema and scales on sun-exposed skin, such as the face, balding scalp, and backs of the hands, among the elderly.

AK develops as a result of chronic ultraviolet (UV) exposure and may lead to invasive squamous cell carcinoma (SCC). Czarnecki *et al*. found that 72% of cutaneous SCCs (150/208) are caused by AK, making it a serious clinical condition^1^. Criscione *et al*. also determined that 65% of all primary SCCs (122/187) arose from lesions previously diagnosed clinically as AK^2^. AK is prevalent in older individuals with fair skin. AK represents an enormous medical burden, and the annual total cost of AK treatment in the USA, where 5.2 million people are affected annually, was estimated at $920 million^3^.

There are several established independent risk factors for AK, including older age, male sex, high cumulative sun exposure, fair skin type, and living near the equator^4^. However, the most important risk factor is exposure to UV radiation, which induces genetic mutations in keratinocytes and promotes tumor cell expansion.

Obesity has been found to be a risk factor for several cancers; the recent worldwide increase in obesity makes this a great concern^5–7^. However, Gerstenblith *et al*. reported a complicated association between BMI and skin cancer, in that risk of basal cell carcinoma increased with increasing height but decreased with increasing weight and body mass index (BMI) in both sexes, even after adjusting for UV radiation susceptibility and exposure^8^. Pothiawala *et al*. reported that obesity appears to be inversely associated with the development of non-melanoma skin cancers in US Caucasians^9^. Consequently, evidence has not been established for an association between obesity and skin cancer. The conflicting results of the association between obesity and AK showed that obesity itself might not be a risk factor for AK. Additional analyses regarding AK and height are needed.

Concerning the medical burden of AK and the possibility of developing squamous cell carcinoma, the risk factors for AK should be evaluated. The association between AK and anthropometric measures has not yet been evaluated, in spite of the known associations between obesity and cancer and between AK and skin cancer. Therefore, we evaluated the associations between anthropometric measures and AK in South Korea, a racially homogenous nation, using a nationally representative dataset with a long-term follow-up period.

## Materials and Methods

### Study design and data sources

This study used two datasets; the Korean Health Examination database and the Korean National Health Insurance (NHI) Service claims database. The Korean Health Examination database was used to select the subjects and obtain information on confounding variables. In Korea, a general health examination is mandatory for local household owners, office employees, and family members over the age of 40 years, either biannually or annually according to occupation. The health examination data include anthropometric measures of the subjects. We used the linked Korean NHI service claims database for the same subjects to evaluate the occurrence of AK.

This was a nationwide population-based retrospective cohort study. The Korean NHI Service claims database contains all claims data for the Korean NHI program, the Korean Medical Aid program, and all other long-term care insurance programs from 2006 to 2015. The Korean NHI program is South Korea’s universal health care system, which provides coverage for the entire population (48,341,311 individuals in 2006 and 51,574,044 individuals in 2015). Many previous studies have proven the value of the Korean NHI Service claims database as a population-based database^10^. The database lists diagnoses by International Classification of Disease, Tenth Revision (ICD-10) codes.

### Study population and definition of actinic keratosis

**Figure 1:**
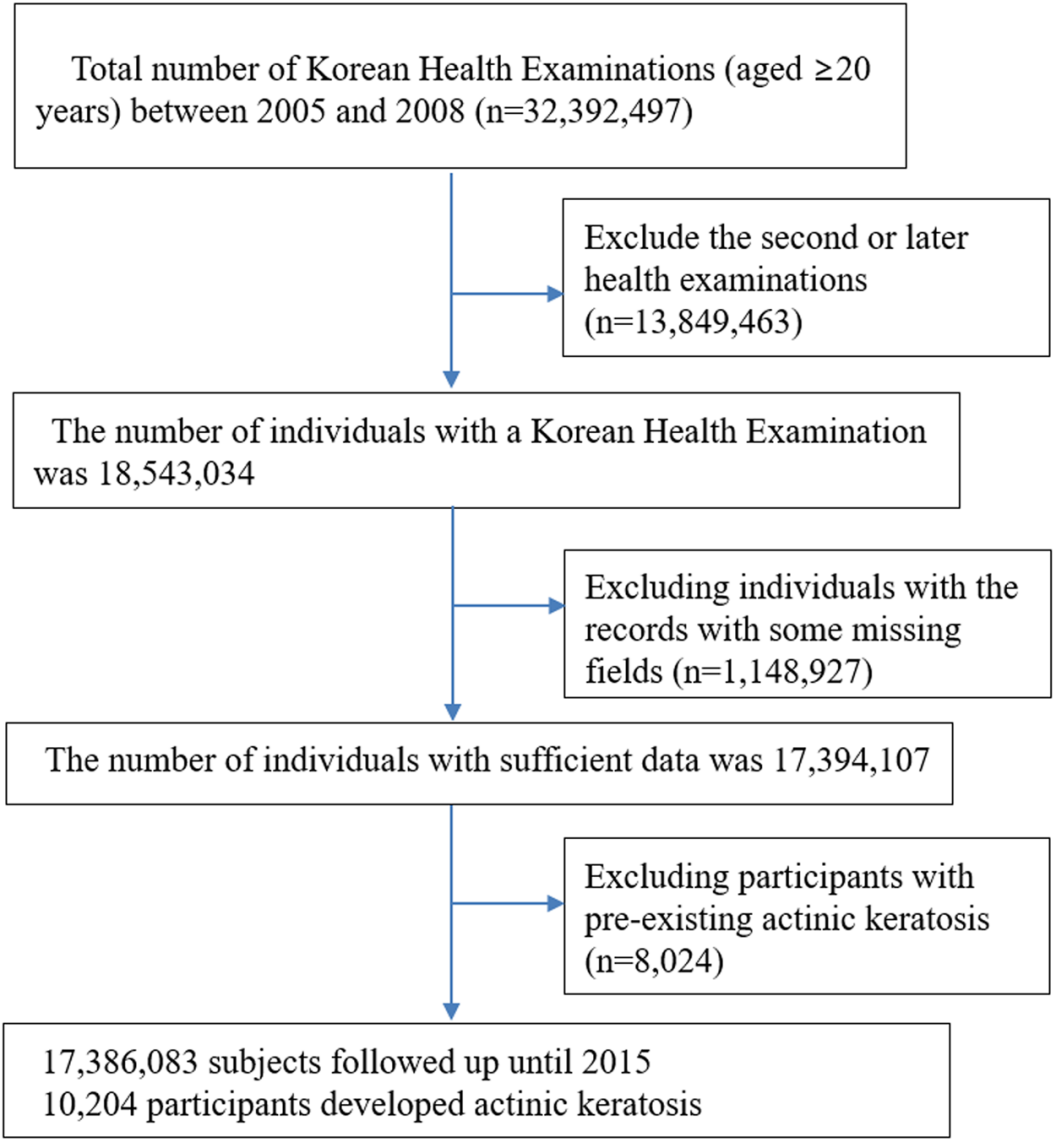
A flow chart of the study.

To find out the association between actinic keratosis and height, we decided to use the Korean Health examination data that provides anthropometric measures and the KNHIS claims database that enables to study the prevalence of AK in Korean population. For using the national database, a customized database was requested from the KNHIS at the beginning of the study. The authors requested a customized database including age, sex, anthropometric measures, a history of comorbid diseases, such as hypertension, dyslipidemia, and diabetes mellitus, and health related behavior including alcohol consumption and smoking habit of each individuals. Although the outdoor activity and exercise are related with sun exposure and considered as important confounding factors, this information was not available. The total number of health examinations in Korean adults (over 20 years of age) conducted between 2005 and 2008 was 32,392,497. If multiple health examinations were performed on a single participant between 2005 and 2008 (n = 13,849,463), only data from the first health examination data were included in the analysis. The year in which subjects first underwent a health examination was considered the index year for that subject. We excluded individuals if they had data missing for any of the measures listed in the section ‘Measurements’ below (n = 1,148,927). After then, we requested KNHIS claims database with diagnosis of AK, ICD-10 code, L570. We observed all subjects who were diagnosed by dermatologists with AK (ICD-10, L570) based on the Korean NHI service claims database during the study period. To avoid the confounding effect of preexisting AK, those who had a history of AK before the index year (n = 8,024) were excluded. Ultimately, the study population consisted of 17,386,083 subjects. This study population was followed from baseline to the date of diagnosis of AK or until December 31, 2015 (Fig. 1). The Institutional Review Board at the Korea Centers for Disease Control and Prevention approved the protocol. The study was also approved by the Institutional Review Board of the Catholic University of Korea (Approval No. KC17ZESI0124) and was conducted according to the principles of the Declaration of Helsinki. Anonymized and de-identified information was used for analyses, and therefore informed consent was not required.

### Measurements

According to the Korean health examination procedures, age at baseline, sex, and anthropometric measures, including height and weight, were analyzed for all subjects at baseline. Anthropometric measurements were performed while the subjects were wearing light clothing. BMI was calculated as the subject’s weight in kilograms divided by the square of the subject’s height in meters. Blood pressure (BP) was measured while the individual was in a seated position after an at least 5-minute rest period. Smoking status, alcohol drinking status, and income level were assessed by questionnaires. Blood samples were drawn after an overnight fast and measured for serum levels of glucose and total cholesterol. The hospitals in which the health examinations were performed were certified by the NHI Service and subject to regular quality control.

### Definition of chronic diseases

Subjects with comorbidities may take medications, such as antihypertensive drugs, thiazide diuretics, and immunosuppressive drugs, that affect AK development. Therefore, adjustments for comorbidities are also needed in the analysis. We used the Korean NHI Service database to identify the comorbidities of the subjects. The presence of diabetes was defined as (1) at least one claim per year for a prescription of antidiabetic medication under ICD-10 codes E10–14 or (2) fasting glucose level ≥7 mmol/L. The presence of hypertension was defined as (1) at least one claim per year for the prescription of an antihypertensive agent under ICD-10 codes I10–I15 or (2) systolic/diastolic BP ≥ 140/90 mmHg L. The presence of dyslipidemia was defined as (1) at least one claim per year for the prescription of an antihyperlipidemic agent under ICD-10 code E78 or (2) total cholesterol ≥6.21 mmol/L. All direct measures were obtained from the health examination database. The presence of chronic obstructive pulmonary disease, myocardial infarction, and congestive heart failure was defined according to the presence of at least one claim per year associated with the ICD-10 codes J41–44, I21–22, and I50, respectively.

### Statistical analysis

All subjects were divided into age- and sex-specific quintiles according to their baseline height (Supplementary Table 1). Income level was dichotomized at the lowest 20%. Since the well-known risk factors for AK are male sex and elderly age, a descriptive analysis of the incidence of AK by sex and age at baseline was performed. Because little is known about the association between anthropometric measures and AK, we used a general approach for the selection of possible confounders including age as a continuous variable and sex. According to the general approach of model selection, we selected Model 3, containing as many confounders as possible, as the subject of primary analysis. The following confounders were chosen. We included weight as a confounder because of previous reports that revealed an association between obesity and AK incidence. The occurrence of AK may also be correlated with the level of income or status of smoking and alcohol consumption. Elderly individuals taking immunosuppressive medications and photosensitizing medications, such as antihypertensive drugs or thiazide diuretics, might have an increased risk of AK. Although comorbidities of AK have not yet been evaluated, we suspect that chronic diseases, such as hypertension, dyslipidemia, chronic obstructive pulmonary disease, myocardial infarction, and congestive heart failure, should be considered as possible confounders and adjusted in the analyses. For the sensitivity analyses, we also used Model 1, which included age and sex, and Model 2, which includes age, sex, and weight as confounders.

Associations between height and risk of AK were analyzed using Cox’s proportional hazards regression models, and the hazard ratio (HR) and 95% confidence interval (CI) for each height quintile, relative to the reference (the lowest quintile), were presented for each of the three models. We also presented the HR and 95% CI according to height deciles after adjusting for the same confounders as in Model 3. To evaluate the robustness of the association between height and risk of AK, we also performed the same analyses separately for males and females and age (<65 years or ≥65 years). The proportional hazard assumptions were checked using the log-log cumulative survival graph and the time-dependent variable Cox model. All statistical analyses were performed using SAS software (ver. 9.4; SAS Institute, Cary, NC, USA).

## Results

### Demographics according to height quintile

The 17,386,083 participants were divided into five groups according to height. The cutoffs for each quintile are shown in Supplementary Table 1. Table 1 shows participant characteristics by height quintile. The data in Table 1 are unadjusted values.

**Table 1:** Demographics of subjects according to height quintile.

	Height				
	Q1	Q2	Q3	Q4	Q5
	N = 3456881	N = 3486750	N = 3635543	N = 3348250	N = 3458659
Mean age (years)	46.7 ± 14.9	46.2 ± 14.7	45.9 ± 14.7	46.1 ± 14.2	45.5 ± 14.5
Sex (male, %)	1890688(54.69)	1921580(55.11)	1835459(50.49)	1764889(52.71)	1836775(53.11)
Diabetes	280180(8.1)	278661(7.99)	279972(7.7)	267857(8)	270785(7.83)
Weight (kg)	57.7 ± 9.7	61.2 ± 10.1	62.9 ± 10.6	65.3 ± 11	69.1 ± 12.2
BMI (kg/cm^2^)	23.7 ± 3.5	23.6 ± 3.2	23.6 ± 3.2	23.5 ± 3.2	23.5 ± 3.2
<18.5	129714(3.75)	134445(3.86)	146995(4.04)	141568(4.23)	158642(4.59)
18.5–23	1363717(39.45)	1380273(39.59)	1463003(40.24)	1346911(40.23)	1405256(40.63)
23–25	842102(24.36)	863506(24.77)	875431(24.08)	817144(24.41)	831645(24.05)
25–30	1005140(29.08)	1001341(28.72)	1035799(28.49)	937796(28.01)	952360(27.54)
≥30	116208(3.36)	107185(3.07)	114315(3.14)	104831(3.13)	110756(3.2)
Lowest income status (<20%)	860433(24.89)	785040(22.51)	779329(21.44)	692275(20.68)	672987(19.46)
Hypertension	939636(27.18)	928900(26.64)	945712(26.01)	878020(26.22)	887800(25.67)
Dyslipidemia	512847(14.84)	506896(14.54)	527046(14.5)	477827(14.27)	471218(13.62)
COPD	403289(11.67)	403459(11.57)	420893(11.58)	386118(11.53)	400229(11.57)
MI	28055(0.81)	28886(0.83)	29665(0.82)	28053(0.84)	29118(0.84)
CHF	52525(1.52)	48109(1.38)	49326(1.36)	43436(1.3)	45385(1.31)
Actinic keratosis (yes)	9280(0.27)	9434(0.27)	10685(0.29)	9684(0.29)	10204(0.3)
Smoking status					
Non	2340810(67.71)	2311632(66.3)	2472708(68.01)	2231012(66.63)	2273229(65.73)
Ex	263089(7.61)	291030(8.35)	292973(8.06)	295956(8.84)	312560(9.04)
Current	852982(24.67)	884088(25.36)	869862(23.93)	821282(24.53)	872870(25.24)
Alcohol consumption (yes)	1565112(45.27)	1652651(47.4)	1692020(46.54)	1606700(47.98)	1691558(48.9)

BMI, body mass index; CHF, congestive heart failure; COPD, chronic obstructive pulmonary disease; MI, myocardial infarction. Data are expressed as mean ± SD or n (%).

Among the five quintile groups, weight showed an increasing trend from Q1 (57.7 ± 9.7 kg) to Q5 (69.1 ± 12.2 kg). Q5 had a higher proportion of underweight participants (BMI < 18.5 kg/cm2, 4.59%) compared to Q1 (3.75%). The percentage of participants in the lowest income category was higher in Q1 (24.89%) compared to Q5 (19.46%).

There were differences in the incidence of comorbid diseases among the five height groups. Q5 had a lower incidence of hypertension, dyslipidemia, and congestive heart failure than Q1; furthermore, this trend was consistent across Q1 to Q5 for hypertension (27.18%, 26.64%, 26.01%, 26.22%, and 25.67%, respectively), dyslipidemia (14.84%, 14.54%, 14.5%, 14.27%, and 13.26%, respectively), and congestive heart failure (1.52%, 1.38%, 1.36%, 1.3%, and 1.31%, respectively). Percentage figures for current smokers and alcohol drinkers (more than once a week) are also shown in Table 1. The incidence of AK increased with height (0.27%, 0.27%, 0.29%, 0.29%, and 0.3%, respectively).

### Risk of actinic keratosis according to age and sex

**Figure 2:**
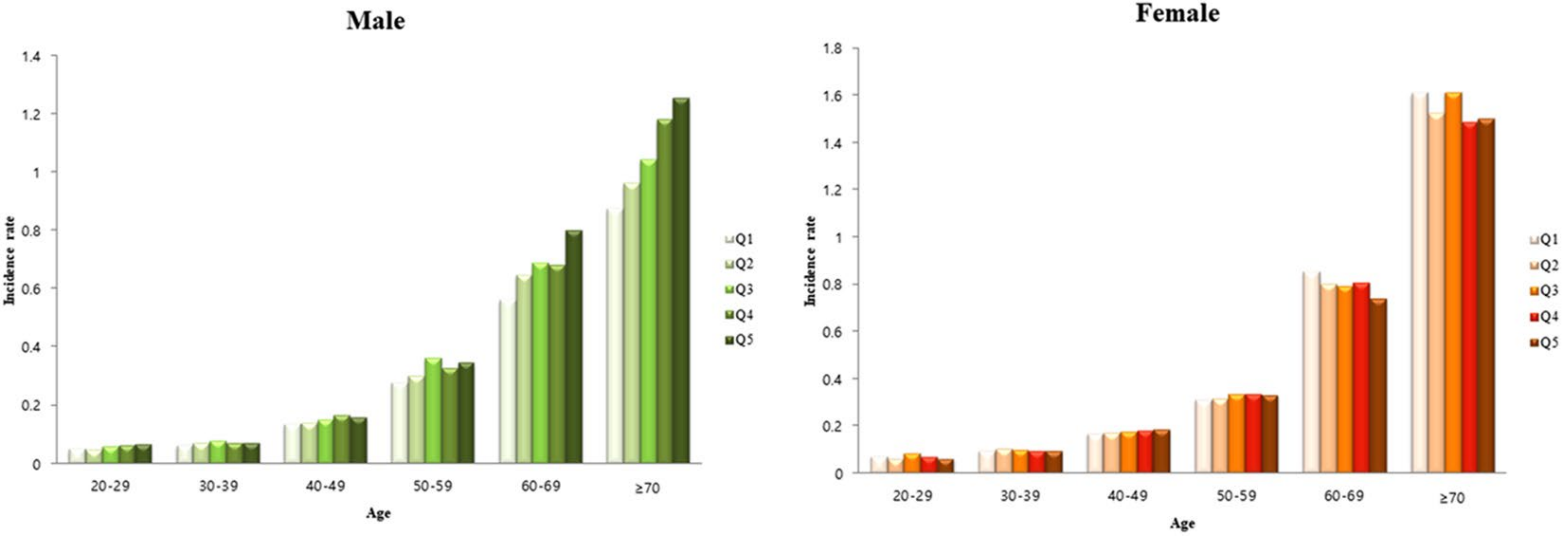
Unadjusted incidence rate of actinic keratosis (AK) according to 10-year age group at baseline, sex and height (in quintiles).

The total number of AK cases was 49,287 and the associated incidence rate was 0.314 per 1000 person-years. The incidence of AK in each group (Q1−Q5) was analyzed according to 10-year age group and sex (Fig. 2). The incidence of AK increased with 10-year age and was highest in those aged over 60 years. In males, the incidence of AK increased with greater height but in females no such association was found.

### Risk of actinic keratosis according to height

**Figure 3:**
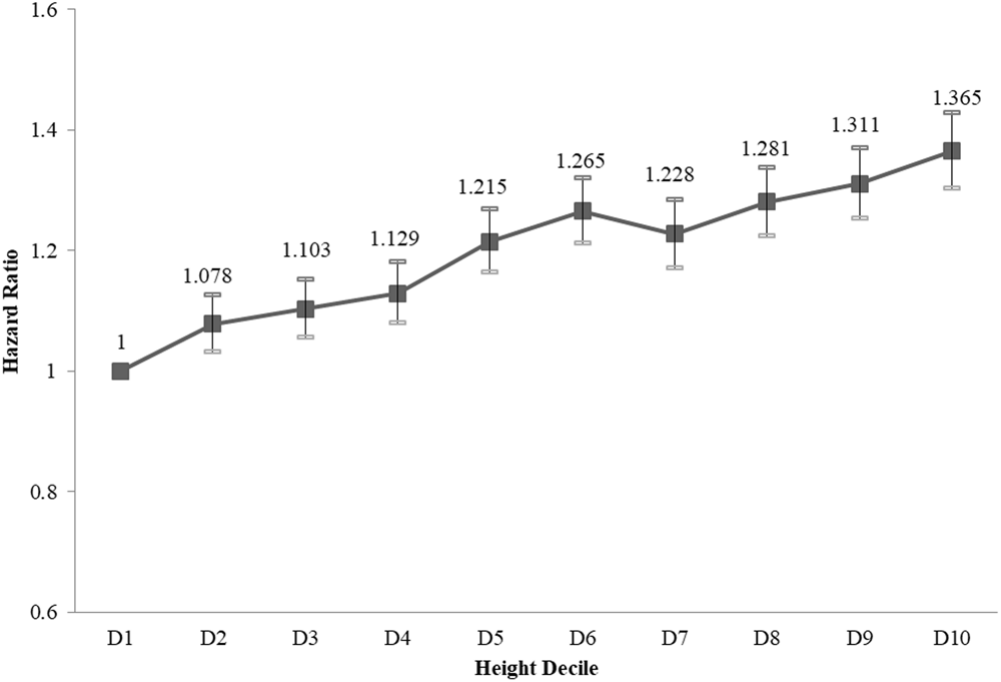
Hazard ratios (HRs) for AK by height decile after adjustment for age, sex, income, smoking status, alcohol consumption, hypertension, dyslipidemia, myocardial infarction, congestive heart failure, and chronic obstructive pulmonary disease. The X-axes show deciles of height, while the Y-axes show hazard ratios with reference to the 1^st^ decile for height. Squares represent HRs, and vertical lines indicate 95% confidence intervals.

Table 2 shows the results of a multivariate analysis of AK risk according to height. Participants in Q5 had a significantly increased risk of AK compared to those in Q1. The risk of AK for taller individuals increased after adjusting for baseline risk factors including age (as a continuous variable), sex, weight, income, smoking, alcohol consumption, hypertension, dyslipidemia, myocardial infarction, congestive heart failure, and chronic obstructive pulmonary disease (Model 3). The estimated risk was especially high for taller males and elderly (≥65 years old) subjects. In females, an association between AK and height became apparent after adjustment for weight. However, adjustment for weight had little impact on the HRs in males. We also presented the HR and 95% CI according to height deciles after adjusting for the same confounders as in Model 3, because *post-ho*c examination of height deciles showed a clearer trend in the HR of AK than the corresponding analysis of height quintiles (Fig. 3). The risk of AK increased with height suggesting that taller individuals are more likely to be diagnosed with AK.

**Table 2:** Multivariate Cox’s proportional hazard regression model of the relationship between height and risk of actinic keratosis.

Group	Number of AK diagnoses	Number of Person-years	Incidence Rate (per 1000 person-years)	Hazard Ratio (95% C.I.)		
				Model 1	Model 2	Model 3
Total						
Q1	9280	31171365.99	0.29771	1	1	1
Q2	9434	31490474.11	0.29958	1.049(1.02,1.08)	1.077(1.046,1.108)	1.073(1.04,1.107)
Q3	10685	32873438.55	0.32503	1.139(1.108,1.171)	1.187(1.154,1.222)	1.193(1.158,1.23)
Q4	9684	30251460.4	0.32012	1.149(1.117,1.183)	1.216(1.181,1.253)	1.207(1.169,1.246)
Q5	10204	31152657.71	0.32755	1.196(1.163,1.23)	1.298(1.259,1.339)	1.284(1.242,1.326)
**Sex**						
Male						
Q1	3639	17104893.96	0.21275	1	1	1
Q2	4175	17391231.84	0.24006	1.122(1.073,1.173)	1.114(1.065,1.165)	1.113(1.061,1.167)
Q3	4243	16672463.43	0.25449	1.249(1.195,1.306)	1.236(1.181,1.293)	1.251(1.193,1.312)
Q4	4284	16013661.68	0.26752	1.284(1.229,1.342)	1.265(1.207,1.325)	1.243(1.182,1.308)
Q5	4642	16625025.02	0.27922	1.402(1.343,1.465)	1.372(1.308,1.44)	1.375(1.306,1.447)
Female						
Q1	5641	14066472.03	0.40102	1	1	1
Q2	5259	14099242.27	0.373	1.006(0.969,1.045)	1.063(1.023,1.104)	1.06(1.017,1.104)
Q3	6442	16200975.12	0.39763	1.069(1.032,1.108)	1.166(1.124,1.209)	1.168(1.122,1.214)
Q4	5400	14237798.72	0.37927	1.063(1.024,1.103)	1.194(1.149,1.242)	1.2(1.151,1.251)
Q5	5562	14527632.68	0.38286	1.066(1.027,1.106)	1.256(1.206,1.308)	1.237(1.184,1.291)
**Age at baseline**						
Age <65						
Q1	3696	24973454.33	0.148	1	1	1
Q2	4110	25648168.6	0.16025	1.068(1.021,1.116)	1.079(1.032,1.128)	1.07(1.019,1.123)
Q3	4494	26393639.07	0.17027	1.174(1.124,1.226)	1.195(1.143,1.249)	1.21(1.155,1.269)
Q4	4406	24727702.99	0.17818	1.178(1.128,1.231)	1.207(1.153,1.263)	1.175(1.118,1.235)
Q5	4271	25217891	0.16936	1.198(1.146,1.252)	1.24(1.183,1.301)	1.222(1.161,1.286)
65 ≥ Age						
Q1	5584	6197911.66	0.90095	1	1	1
Q2	5324	5842305.52	0.91128	1.037(0.999,1.077)	1.084(1.044,1.126)	1.084(1.041,1.129)
Q3	6191	6479799.49	0.95543	1.104(1.064,1.144)	1.185(1.141,1.23)	1.187(1.14,1.235)
Q4	5278	5523757.41	0.95551	1.119(1.078,1.162)	1.233(1.185,1.283)	1.239(1.188,1.292)
Q5	5933	5934766.7	0.9997	1.178(1.136,1.222)	1.352(1.298,1.407)	1.341(1.285,1.4)

Model 1 Adjusted for age and sex.

Model 2 Adjusted for age, sex, and weight.

Model 3 Adjusted for age, sex, weight, income, smoking status, alcohol consumption, hypertension, dyslipidemia, myocardial infarction, congestive heart failure, and chronic obstructive pulmonary disease.

## Discussion

We found that greater height was associated with increased risk of AK among a South Korean population. This suggests that anthropometric characteristics might have a role in the pathogenesis of AK related to the genetic factors of height. Although height is determined by both genetic and environmental factors, the influence of genetic factors on height is estimated to be approximately 60% to 84%^11–13^. The genetic influence on height is reported to be very similar among different populations in spite of substantial variations in mean height^14^. The importance of genetic factors in determining the height of South Korean children was demonstrated by a previous study^15^.

In this study, greater height was significantly associated with AK. The tallest group (Q5) had a higher risk than the shortest group (Q1). We revealed the results of Models 1, 2, and 3 were almost identical (Table 2). As shown in the Fig. 2, incidence of AK had the tendency to increase with age, therefore we adjusted for age as a continuous variable. The other confounders in this study were sex, weight, health-related behaviors (smoking and drinking), income status, and comorbid chronic diseases such as hypertension, dyslipidemia, congestive heart failure, chronic obstructive pulmonary disease, and history of myocardial infarction (Model 3).

An association between height and heart disease has been reported in several studies. Goldbourt *et al*. demonstrated that the relative risk associated with being in the shortest versus the tallest quartile was 1.54 (95% CI: 1.13, 2.10)^16^. Silventoinen *et al*. also reported an inverse association between height and heart disease^17^. Bjornsson *et al*. recently reported that shorter stature is associated with a greater degree of coronary atherosclerosis in a large, unselected population of individuals undergoing coronary angiography^18^. In the present study, increased incidences of dyslipidemia, congestive heart disease, and hypertension were observed in shorter versus taller participants; this could also be explained by reference to the genetic and environmental factors that influence human height.

This study is the first to find a relationship between height and the incidence of AK. One of the major strengths of this study was the inclusion of a large study population, made possible by use of the Korean NHIS Claims Database, which contains all claims data for the Korean NHI program and the Korean Medical Aid program. The Korean NHIS covers almost 99% of the Korean population. Therefore, the Korean NHIS Claims Database has a sample size of over 50 million individuals^19^. The database includes AK patients with pathologic diagnoses. In South Korea, AK is diagnosed by a dermatologist after pathologic examination. In Korea, there are no dermatologists outside of the KNHIS, since they should ask the service to insure. Another strength concerns the racial homogeneity of the South Korean population, which minimized bias due to racial differences.

One limitation of this study was that the progression of AK to non-melanoma skin cancer was not investigated. Second, it was not possible to include all patients with AK, because patients with AK might not have visited a dermatologic clinic. Third, sun exposure and occupational information are not available in the Korean NHI Claims Database. UV radiation is the most important risk factor for AK development. Without evaluating sun exposure, it is difficult to determine the impact of height on the development of AK. There may be a greater tendency for participants with lower BMI and greater height to spend time outdoors. Finally, this study could not include all the patients with actinic keratosis. This study enrolled subjects who had undergone a national health examination; the number of participants (17 million) was approximately the half of the Korean population over 20 years of age (37 million). The presence of actinic keratosis was obtained from the KNHIS. For considering that the unmet medical care needs is around 9% and people with unmet health care needs tend to be the elderly and rural residents^20^, the actual incidence of AK may be higher than that of this study. The results of this study could be generalized to the majority of Koreans who receive a medical examination. However, it is unclear whether the results can be generalized to Koreans who have not received an examination or to populations in other countries.

This study demonstrated that AK was significantly associated with greater height. Further studies of the potential mechanisms underlying the association between height and AK incidence are needed.

## Electronic supplementary material

Supplementary table 1.
