# Supplementary material for: Association between Height and Actinic Keratosis: A Nationwide Population-based Study in South Korea
Source: Sci Rep. 2018 Jul 18;8:10897. doi: 10.1038/s41598-018-29155-6 (PMC6052058; doi:10.1038/s41598-018-29155-6)
Supplement: Supplementary file 1 — Supplementary table 1. [file 41598_2018_29155_MOESM1_ESM.doc]

Original article

**Association between Height and Actinic Keratosis: A Nationwide Population-based Study in South Korea**

Young Bok Lee^1^, Ji Hyun Lee^1*^, Min Ji Kang^1^, Jin-Wou Kim^1^, Dong Soo Yu^1^, Kyung Do Han^2^, Yong Gyu Park^2*^.

^1^Department of Dermatology, College of Medicine, The Catholic University of Korea, Seoul, Korea

^2^Department of Biostatistics, College of Medicine, The Catholic University of Korea, Seoul, Korea

Correspondence and reprint requests should be addressed to: Yong Gyu Park, Ph.D., Department of Biostatistics, College of Medicine, The Catholic University of Korea, 222, Banpo-daero, Seocho-gu, Seoul, 137-701, Korea, e-mail: ygpark@catholic.ac.kr, Ji Hyun Lee. M.D., Ph.D. Department of Dermatology, College of Medicine, The Catholic University of Korea, 222, Banpo-daero, Seocho-gu, Seoul, 137-701, Korea, Tel: 82-31-820-3509, Fax: 82-31-846-4799, e-mail: yiji1@hanmail.net

*Ji Hyun Lee and Yong Gyu Park equally contributed equally this work.

Supplementary table 1. Table that illustrates the height of subjects according to age, sex and.height quintile (cm).

| **Height** |  |  |  |  |  |  |  |  |
| --- | --- | --- | --- | --- | --- | --- | --- | --- |
| **Age (years)** | SEX | number of individuals | Minimum | 1^st^ quintile | 2^nd^ quintile | 3^rd^ quintile | 4^th^ quintile | Maximum |
| **20-29** | male | 1447243 | 160 | 169 | 172 | 175 | 178 | 208 |
|  | female | 1363786 | 149 | 157 | 160 | 162 | 165 | 198 |
| **30-39** | male | 2346926 | 159 | 167 | 171 | 173 | 177 | 207 |
|  | female | 984444 | 148 | 155 | 158 | 161 | 164 | 197 |
| **40-49** | male | 2198356 | 157 | 165 | 168 | 171 | 174 | 202 |
|  | female | 2100986 | 145 | 153 | 156 | 158 | 161 | 199 |
| **50-59** | male | 1655368 | 154 | 163 | 166 | 169 | 172 | 199 |
|  | female | 1793166 | 143 | 151 | 154 | 157 | 160 | 204 |
| **60-69** | male | 1062185 | 152 | 161 | 164 | 167 | 170 | 198 |
|  | female | 1179594 | 140 | 148 | 151 | 154 | 157 | 198 |
| **>70** | male | 539313 | 159 | 159 | 162 | 165 | 168 | 199 |
|  | female | 714716 | 135 | 144 | 148 | 151 | 154 | 197 |
